# Cloning, Expression, Invasion, and Immunological Reactivity of a Mammalian Cell Entry Protein Encoded by the mce1 Operon of *Nocardia farcinica*

**DOI:** 10.3389/fmicb.2017.00281

**Published:** 2017-02-22

**Authors:** Xingzhao Ji, Xiaoluo Tan, Xuexin Hou, Chenchen Si, Shuai Xu, Lu Tang, Xiuqin Yuan, Zhenjun Li

**Affiliations:** ^1^State Key Laboratory of Infectious Disease Prevention and Control, National Institute for Communicable Disease Control and Prevention, Chinese Center for Disease Control and PreventionBeijing, China; ^2^Office of Emergency Response, Chenzhou Center for Disease Control and PreventionChenzhou, China; ^3^School of Public Health, University of South ChinaHengyang, China

**Keywords:** nocardiosis, Mce1E, entry protein, IFN-γ, recombinant protein expression

## Abstract

Bacterial mammalian cell entry (Mce) proteins have been implicated in pathogen invasion of mammalian host cells. The aim of this study was to examine the invasion-conferring ability of *mce1E* operon-encoded proteins, *in vivo* expression of Mce1E in cells from infected mice and rabbits, and Mce1E immunogenicity. *Nocardia farcinica mce1E* was cloned into pet30a(+) vectors, expressed in *Escherichia coli*, and purified. Invasion assays, transmission electron microscopy (TEM), immunoblots, and enzyme-linked immunosorbent assay (ELISA) detection of cytokines were conducted. TEM confirmed the invasion of HeLa cells by Mce1E-coated beads. The antigenicity of *E. coli*-expressed recombinant Mce1E was confirmed in immunoblots with sera from *N. farcinica*-infected mouse and rabbit sera. Co-incubation of Mce1E with splenocytes of *N. farcinica*-infected mice demonstrated upregulation of interferon (IFN-γ), but not interleukin (IL)-4 or IL-10, in the cultural supernatant. These findings demonstrate that Mce1E may facilitate *N. farcinica* interactions with and invasion of mammalian cells. Notably, Mce1E are expressed and elicited antibody responses in mice and rabbits during infection. Besides, it may play a role in cell-mediated immune reactions and cause host inflammation responses to *N. farcinica* infection.

## Introduction

*Nocardia* genus bacteria are Gram-positive filamentous rod, aerobic pathogens found in soil and water worldwide (Scharfen et al., 2010). They are considered opportunistic pathogens, affecting predominantly immunocompromised patients, including patients with AIDS and transplant recipients (Kim et al., 2016). Pulmonary disease is the most common presentation of Nocardia in immunosuppressed patients and approximately one-third of affected patients have a disseminated disease (Ambrosioni et al., 2010; Kandi, 2015; Scorey and Daniel, 2016). Infection of traumatic wounds produces chronic inflammation that may lead to fistulas, abscesses, cellulitis, ulcerations, and mycetoma (Smego and Gallis, 1984; Salinas-Carmona, 2000; Salinas-Carmona et al., 2009), and may extend into muscles, bones, the brain, kidneys, the prostate, cornea, heart, and adjacent organs (De Nardo et al., 2013; Sirijatuphat et al., 2013; Kumar et al., 2014; Park et al., 2014; Sharma and O’Hagan, 2016). *Nocordia* infection of the central nervous system may be acquired by cutaneous or respiratory routes (Smego and Gallis, 1984; Beaman and Beaman, 1994; Inamadar and Palit, 2003; Zakaria et al., 2008; Chen et al., 2016).

The incidence of *Nocardia* infection cases has been increasing in recent years. Thus far, some 25 *Nocordia* species have been found to infect human patients, including *Nocordia brasiliensis, N. asteroides, N. farcinica, N. abscessus, N. nova*, and *N. transvalensis complex*; among them, *N. farcinica* is the most commonly encountered species (Kandi, 2015). Clinically, untreated pulmonary nocardiosis resembles tuberculosis and thus represents a risk for misdiagnosis (Ekrami et al., 2014).

The mechanisms of *Nocardia*-macrophage interaction have not been resolved. To achieve infection, bacteria must first escape host defenses. Bacteria can enter human macrophages through interactions with cell-surface receptors (Glickman and Jacobs, 2001). The mammalian cell entry (Mce) proteins, encoded by *mce* genes, are a family of invasion proteins expressed by Mycobacteria. They have putative signal sequences at the N-terminus and are thought to be localized to the mycobacterial cell surface (Ahmad et al., 2005). Mce protein expression in non-pathogenic *Escherichia coli* has been shown to enable the bacteria to enter and survive within HeLa cells and macrophages (Arruda et al., 1993; El-Shazly et al., 2007; Saini et al., 2008). Six *mce* operons were identified in *N. farcinica* (Ishikawa et al., 2004). However, no prior study has clarified whether the Mce1E protein in *N. farcinica* enables host cell invasion. Furthermore, the immunological reactivity of Mce1E has not been described in the literature.

In the present study, we examined whether expression of purified recombinant *N. farcinica* Mce1E protein can promote *N. farcinica* invasion of mammalian cells. Additionally, we assessed expression of Mce1E in *N. farcinica* infections. Finally, we explored Mce1E immunogenicity in murine splenocytes infected with *N. farcinica*.

## Materials and Methods

### Ethics Statement

Laboratory animal care and experimentation were performed in accordance with animal ethics guidelines and approved protocols. The animal experiments were approved by the Ethics Review Committee of the National Institute for Communicable Disease Control and Prevention at the Chinese Center for Disease Control and Prevention.

### Bacterial Strains, Plasmid, and Anti-*N. farcinica* Sera

Standard DSM43131 strain *N. farcinica* bacteria were purchased from the German Resource Centre for Biological Materials and grown in brain-heart-infusion medium at 37°C (Difco Laboratories Inc., Detroit, MI, USA). The pET30a(+) plasmid was used as an expression vector and *E. coli* BL21 (DE3) were used a host for the vector, as recommended by the manufacturer. *E. coli* colonies (TransGen Biotech, China) were grown in Luria-Bertani (LB) medium at 37°C. Anti-*N. farcinica* sera were prepared from BALB/c mice and New Zealand rabbits in our laboratory.

### Expression and Purification of Recombinant Mce1E

The *mce1E* gene was amplified from *N. farcinica* genomic DNA by polymerase chain reaction with the following specific primers: forward 5′-GTA T**CA TAT G**AT GAG ACG CGC GCG TCG CAC-3′ and reverse 5′-GAT C**AA GCT T**TC GGC CCT GTC CCC CCT CGA-3′. Polymerase chain reaction products were digested by Nde I and Hind III and then introduced into the pET-30a(+) prokaryotic expression vector. The recombinant plasmids were sequenced and then transformed into *E. coli* BL21 cells for fusion protein expression. The *E. coli* BL21 cells were cultured at 37°C with agitation in LB medium containing 50 μg/ml kanamycin until their optical density at 600 nm reached 0.8. Subsequently, the cells were induced with 1 mM isopropyl β-D-1-thiogalactopyranoside (IPTG) at 30°C for 6 h.

After sonication and centrifugation, Mce1E protein molecules were solubilized in binding buffer containing 6 M urea. The solubilized proteins were chromatographed on a His column in accordance with the manufacturer’s instructions (Novagen, Germany). Purified protein were dialyzed in a concentration gradient of urea (6, 4, 2, and 1 M) to allow renaturing at 4°C for 24 h. The renatured proteins were placed in phosphate-buffered saline (PBS) overnight at 4°C. The recombinant Mce1E proteins were examined by sodium dodecyl sulfate polyacrylamide gel electrophoresis (SDS-PAGE) and their concentrations were determined with a BCA protein assay kit (Thermo Scientific, USA).

### Coating of Beads with Recombinant Proteins and HeLa Cell Culture

A 5-μl sample of stock latex bead suspension (4%w/v, 0.3-μm diameter, Thermo Fisher) was mixed with 1 ml PBS containing 60 μg Mce1E protein; uncoated latex beads served as the control treatment. Samples from all groups were incubated for 2 h at 37°C. HeLa cells were cultured with Dulbecco’s modified Eagle’s medium (Gibco) supplemented with 10% fetal calf serum (FCS; Gibco) at 37°C.

### Invasion Assays and Transmission Electron Microscopy (TEM) Analysis

HeLa cells were harvested, washed, and re-suspended in Dulbecco’s modified Eagle’s medium. Subsequently, they were seeded in a 24-well polystyrenetissue culture plates and incubated to form monolayers of cells. MceIE-coated latex beads and uncoated latex beads (200 μl per aliquot) were added to near-confluent HeLa cell monolayers grown in 24-well plates. The cells were incubated at 37°C for 24 h in a CO_2_ incubator, and then washed three times with PBS. The washed cells were fixed in 2% glutaraldehyde, postfixed in 1% osmium tetroxide, and dehydrated through increasing grades of ethanol solution. Samples of cells were embedded, and then cut into ultrathin sections. The ultrathin sections were stained and examined by transmission electron microscopy (TEM) with a microscope HT7700 (Japan).

### Western Blot

To confirm recombinant protein expression, proteins were separated by SDS-PAGE (5–12%) and transferred onto polyvinylidene fluoride membranes with 100 V for 1 h. The membranes were then blocked with blocking buffer (5% skim milk in PBS, pH 7.4, with 0.05% Tween 20) overnight at 4°C. A 1:4000 dilution of a horse-radish peroxidase (HRP)-conjugated monoclonal anti-pentahistidine (His) antibody (New England Biolabs Inc., USA) was applied to the membranes for 1 h to detectthe His tag. Mouse and rabbit antisera (1:2000) were used as primary antibodies and an IgG-HRP goat antibody (Sigma) was used as secondary antibody to detect recombinant Mce1E. Detection was performed with a diaminobenzidine kit (DAB, Tiangen).

### Spleen Cell Preparation and Cytokine Detection

A unicellular suspension containing 10^8^ colony forming units per ml of *N. farcinica* in the log phase of growth was injected subcutaneously (500 μl) into BALB/c mice (6–8 weeks). Spleens were harvested 1, 3, 7, and 14 days after infection (*N* = 3 per time point). The red blood cells were removed from the spleen samples and the spleen lymphocytes were washed and then suspended at a concentration of 1 × 10^6^ cells per ml of RPMI-1640 medium with 10% FCS. Cultures containing 1 × 10^6^ cells were stimulated with 1 μg/ml of recombinant Mce1E or phytohemagglutinin A (PHA). The stimulated cultures were incubated for 24 h in 5% CO_2_ at 37°C with constant humidity (95%). Subsequently, the cultures were centrifuged at 10000 rpm for 5 min to remove the cells. Interferon (IFN)-γ, interleukin (IL)-10, and IL-4 concentrations in the supernatant were determined by quantitative enzyme-linked immunosorbent assay (ELISA; IFN-γ ELISA kit from RD, IL-10 and IL-4 ELISA kits from BD, USA). All cultures were processed in triplicate.

### Statistical Analysis

Group means and standard deviations (SDs) were compared with Student’s *t*-tests. Differences were considered statistically significant when *p*-values were below 0.05.

## Results

### Expression and Purification of Recombinant Mce1E

**FIGURE 1:**
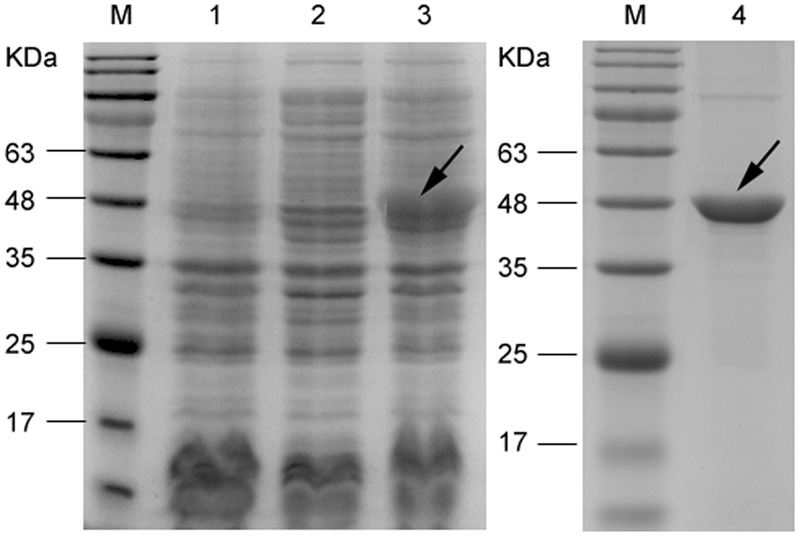
**Sodium dodecyl sulfate polyacrylamide gel electrophoresis (SDS-PAGE) analysis of recombinant Mce1E expressed in *Escherichia coli* BL21.** Lane M, marker; lane 1, vector control (whole-cell protein); lane 2, uninduced control (whole-cell protein); lane 3, induced (30°C for 6 h; whole-cell protein); and lane 4, purified Mce1E protein.

Nucleotide sequencing analysis confirmed that *mce1E* had been inserted correctly into the pet30a vector. As shown in **Figure 1**, SDS-PAGE results showed that after cells were induced with 1 mM IPTG (30°C for 6 h), they exhibited increased expression of a 48-kDa protein. It was further demonstrated that purification removed, to a large extent, other proteins, leaving a predominant band at 48 kDa.

### Invasion of HeLa Cells by mce1E-Coated Latex Beads

**FIGURE 2:**
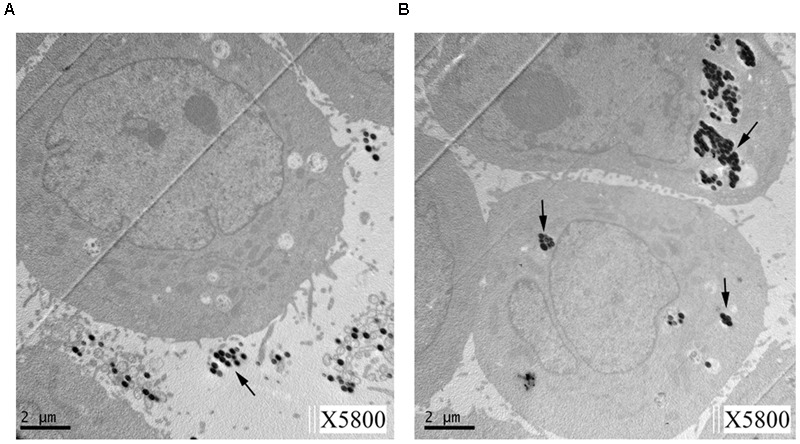
**Transmission electron microscopy (TEM) of HeLa cells showing internalization of Mce1E-coated beads. (A)** Non-coated beads were not observed in HeLa cells. **(B)** Mce1E-coated beads were completely internalized by HeLa cells. The beads were present in HeLa cells either alone or in clusters.

As shown in **Figure 2**, TEM confirmed that beads coated with Mce1E entered HeLa cells. Internalized coated-beads were observed within vacuolar compartments after 24 h of incubation; non-coated beads were not observed within vacuolar compartments after 24 h of incubation.

### Putative Mce1E Protein Band Bound Specifically by Sera from *N. farcinica*-Infected Animals

**FIGURE 3:**
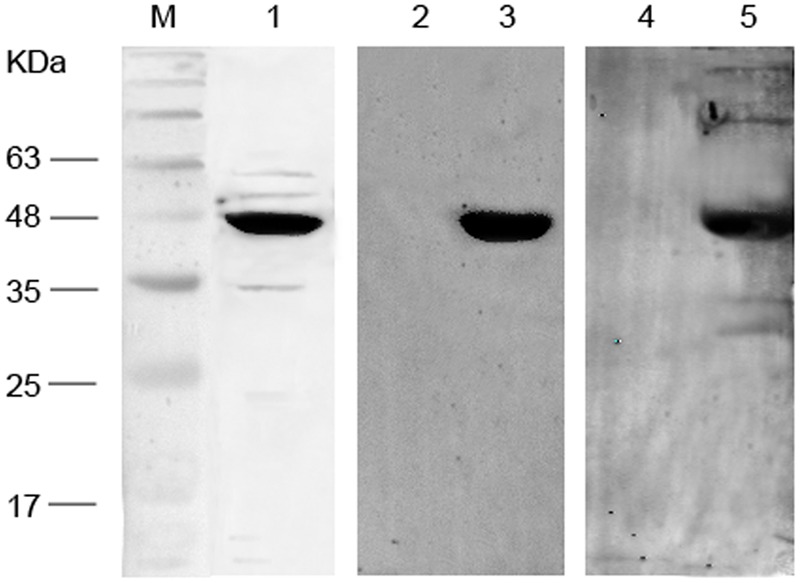
**Western blot analysis of recombinant Mce1E.** Lane M: Marker; lane1, purified Mce1E immunoblotted with anti-His antibodies; lane 2, purified Mce1E immunoblotted with sera from BALB/c mice infected with normal saline; lane 3, purified Mce1E immunoblotted with sera from BALB/c mice infected with *Nocardia farcinica*; lane 4, purified Mce1E immunoblotted with sera from New Zealand white rabbits infected with normal saline; lane5, purified Mce1E immunoblotted with sera from New Zealand white rabbits infected with *N. farcinica*.

As shown in **Figure 3**, expressed 48-kDa fusion proteins were detected in immunoblots with a major band of reactivity at the expected migration positions for the anti-His antibody employed. Importantly, the 48-kDa immunopositive band was also observed when purified Mce1E was immunoblotted with sera from *N. farcinica*-infected mice and rabbits. No bands were detected at 48 kDa when purified Mce1E was immunoblotted with control mouse or rabbit sera.

### Cytokine Detection

**FIGURE 4:**
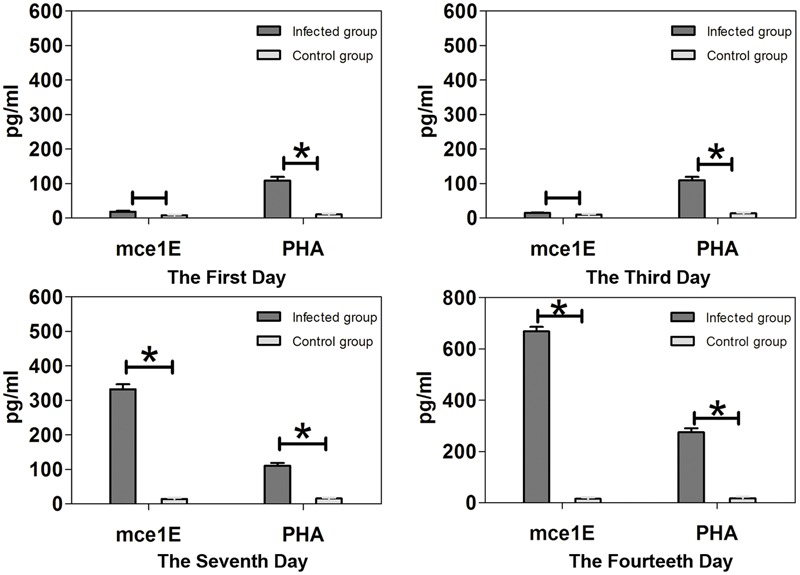
**Interferon-γ production by splenocytes stimulated with Mce1E or PHA.** Upregulated production of IFN-γ was detected in Mce1E-stimulated cultures of cells from *N. farcinica*-infected mice 7 and 14 days, but not 1 or 3 days, after infection. ^∗^*P* < 0.05.

Spleen cell IFN-γ production was increased by Mce1E stimulation 7 and 14 days after *N. farcinica* infection (both *p* < 0.05) and this IFN-γ production increased with the progression of time (**Figure 4**). Spleen cell production of IL-4 and IL-10 remained low following stimulation with recombinant Mce1E protein, with no statistically significant differences among the time-point groups (data not shown).

## Discussion

In the present study, we obtained data confirming the ability of *N. farcinicaon* Mce1E to enable invasion of mammalian cells. We further confirmed that the recombinant Mce1E that produced internationalization of associated beads was immunologically reactive. These data suggest that *N. farcinicaon* Mce1E is functionally similar to *Mycobacterium tuberculosis* Mce proteins, which enable *M. tuberculosis* mammalian-cell invasion, and thus pathogenesis, leading to long-term survival and proliferation of the pathogenic bacteria in host cells (Gioffre et al., 2005). The present work extends the work of Arruda et al. (1993) who showed that Mce1E can confer upon non-pathogenic *E. coli* the ability to invade HeLa cells, escape host defenses, augment macrophage phagocytosis, and survive for at least 24 h in human macrophages.

In Mycobacteria, as well as five other Actinomycetales genera and some Gram-negative bacteria, *mce* operons are widely distributed but structurally identical (Casali and Riley, 2007). The pathogenicity of these factors might be determined by their expression (Haile et al., 2002; Shimono et al., 2003; Casali and Riley, 2007). Mce3A, Mce3D, and Mce3E—encoded by the *mce3* operon*—*are expressed by *M. tuberculosis* and elicit antibody responses in a majority of naturally infected human patients (Ahmad et al., 2004, 2005). Indeed, Mce1A and Mce1E have been found in the sera of tuberculosis patients (Ahmad et al., 1999). Meanwhile, it was reported recently that *N. brasiliensis* HUJEG-1 possesses 33 *mce* genes distributed in six operons (Vera-Cabrera et al., 2013). Moreover, *N. nova* SH22a was found to have significantly more *mce* clusters than *N. farcinica* IFM 10152 or *N. brasiliensis* HUJEG-1 (Luo et al., 2014). Notwithstanding, *Nocardia* Mce proteins have been given scant attention despite the fact that the corresponding proteins are considered to be an important virulence factor in *M. tuberculosis* (Arruda et al., 1993).

Recombinant *M. bovis* Mce4A and Mce4E have been shown previously to stimulate alveolar macrophages, there by upregulating the expression of tumor necrosis factor-alpha, inducible nitric oxide synthase, and IL-6, without affecting IL-12 (Xu et al., 2007, 2008), suggesting that these proteins may cause host inflammation responses to *M. bovis* infection. Conversely, Li et al. (2015) showed that *M. tuberculosis* Mce3E can suppress host innate immune responses by inhibiting activation of the extracellular signal-regulated kinase 1/2 signaling pathway and suppressing expression of tumor necrosis factor and IL-6. Here, we found that our recombinant *N. farcinica* Mce1E can stimulate spleen lymphocytes of *N. farcinica*-infected mice to express IFN-γ, while IL-4 and IL-10 were not detected. The steady increase in lymphocyte production of IFN-γ from 3 days post-infection onward may be explained by *N. farcinica* achieving entry of macrophages 3 days after infection.

## Conclusion

The present data showed that Mce1E encoded by the *mce1* operon of the *N. farcinica* genome facilitated internalization of latex beads by non-phagocytic mammalian (HeLa) cells. Furthermore, we found that Mce1E was expressed by *N. farcinica* in the process of infection in animals, suggesting it may also be expressed in humans with *N. farcinica* infections. Our results showing that Mce1E can stimulate spleen lymphocytes of *N. farcinica*-infected mice to express IFN-γ suggest that mce1E play a role in cell-mediated immune reactions.

## Author Contributions

XJ, XT, ZL, and XY conceived and designed the experiments. XJ and XT wrote the manuscript. XJ and XT performed the experiments. XJ and XT analyzed the data. XH, CS, SX, and LT contributed reagents/materials/analysis tools. ZL supported financially and administratively, final approval of manuscript.

## Conflict of Interest Statement

The authors declare that the research was conducted in the absence of any commercial or financial relationships that could be construed as a potential conflict of interest.
